# Theoretical Analysis of Stacking Fault Energy, Elastic Properties, Electronic Properties, and Work Function of Mn_x_CoCrFeNi High-Entropy Alloy

**DOI:** 10.3390/ma17174378

**Published:** 2024-09-04

**Authors:** Fenger Sun, Guowei Zhang, Hong Xu, Dongyang Li, Yizheng Fu

**Affiliations:** 1 School of Intelligent Manufacturing Industry, Shanxi University of Electronic Science and Technology, Linfen 041000, China; 2 School of Material Science and Engineering, North University of China, Taiyuan 030051, China; 3 Department of Chemical and Materials Engineering, University of Alberta, Edmonton, AB T6G 1H9, Canada

**Keywords:** high-entropy alloy, generalized stacking fault energy, first-principle, elastic property, electronic property, work function

## Abstract

The effects of different Mn concentrations on the generalized stacking fault energies (GSFE) and elastic properties of Mn_x_CoCrFeNi high-entropy alloys (HEAs) have been studied via first-principles, which are based on density functional theory. The relationship of different Mn concentrations with the chemical bond and surface activity of Mn_x_CoCrFeNi HEAs are discussed from the perspectives of electronic structure and work function. The results show that the plastic deformation of Mn_x_CoCrFeNi HEAs can be controlled via dislocation-mediated slip. But with the increase in Mn concentration, mechanical micro twinning can still be formed. The deformation resistance, shear resistance, and stiffness of Mn_x_CoCrFeNi HEAs increase with the enhancement of Mn content. Accordingly, in the case of increased Mn concentration, the weakening of atomic bonds in Mn_x_CoCrFeNi HEAs leads to the increase in alloy instability, which improves the possibility of dislocation.

## 1. Introduction

High-entropy alloys (HEAs), also known as multicomponent alloys, are alloys consisting of five or more equal or approximately equal amounts of metals. The concentration of each element in the alloy is between 5 and 35% [1,2]. It has a high entropy effect, extreme lattice distortion effect, cocktail effect, and sluggish diffusion effect [3,4,5,6]. In recent years, more and more people have paid attention to high-entropy alloys because of their unique compositions and excellent properties [7,8,9,10]. In particular, the high-entropy alloy Fe_20_Cr_20_Mn_20_Ni_20_Co_20_, with equal proportions, also known as the Cantor alloy, was first introduced in 2004 [11]. Therefore, this kind of CrMnFeCoNi alloy has long been a focus and hotspot of research.

So far, many researchers have extensively studied CoCrFeNi-based high-entropy alloys. Li et al. [12] designed a kind of metastable high-entropy dual-phase alloy, Fe_80-x_Mn_x_Co_10_Cr_10_. They concluded that the single face-centered cubic (FCC) phase structure could be satisfied when the Mn content is 40 and 45% (Fe_35_Mn_45_Co_10_Cr_10_ and Fe_40_Mn_40_Co_10_Cr_10_, respectively). The Mn content plays an important role in phase composition, regulating phase stability and improving phase transition mechanism. Sun et al. [13] compared the lattice stability of Al_x_CrMnFeCoNi and Al_x_CrFeCoNi high-entropy alloys. It was found that Mn decreases the stable field of FCC phase and widens the width of the two-phase region. Moreover, via the exact muffin-tin orbitals (EMTO) method, Zhang et al. [14] calculated the elastic properties of body-centered cubic (BCC) and face-centered cubic (FCC) Al_x_CrMnFeCoNi (0 ≤ x ≤ 5) HEAs, demonstrating that there is a complex dependence between the elastic parameters and the composition, and the elastic anisotropy of both phases is extremely high. Shi Y.Z. et al. [15] studied the homogenization effect of 1250 °C heat treatment on Al_x_CoCrFeNi HEAs. After heat treatment, the homogenization effect of microstructure leads to the decrease in work function and the improvement of corrosion resistance. Zhang et al. [16] prepared CoCrFeNi-Nb_x_ (x = 0, 1, 3, 5, 7, 9 wt%) high-entropy alloys through high-energy ball milling and discharge plasma sintering. The effects of niobium on the microstructure and properties of cobalt–nickel alloys were studied systematically. Nb atoms cause lattice distortion of the alloy, and the microstructure of CoCrFeNi HEAs changes from a single-phase structure of FCC to a bi-phase structure of FCC and Laves, which increases the tensile strength, yield strength, and hardness of the HEA.

Moreover, Kivy M.B. et al. [17] investigated the effects of Cu, Mn, Al, Ti, and Mo on the generalized stacking fault energies (GSFE), Rice-criterion ductilities, and twin ability of CoCrFeNi-based HEAs with FCC structure via density functional theory. The results presented that the addition of Ti and Mo increases the tendency of dislocation slip and deformation twinning. Furthermore, the addition of Mn, Cu, or Al with high content promoted dislocation slip and martensitic phase transition, while a low amount of Al led to dislocation slip. Achmad et al. [18] have calculated pure cobalt and Co-9 at.% X solid-solution alloys (X = Cr, W, Mo, Ni, Mn, Al, Fe) using first-principles density-functional theory. They found that the alloying of Cr, W, and Mo increases the interlayer distance distortion of the stacking fault plane and reduces the GSFE value of pure Co due to the increase in charge accumulation. Also, the stacking fault energy (SFE) values of several typical FCC high-entropy alloys (HEAs) were measured by Liu et al. [19] using experimental means. The experimental results indicated that the lower SFE is helpful for the formation of deformation twins under the loading condition, and the smaller the thickness is, the better the mechanical properties are at low temperature.

It is obvious from the above research results that the Mn element plays an important role in the CoCrFeNi-based high-entropy alloy. Although a great deal of research has been conducted on the effects of alloying elements, especially Mn, on high-entropy alloys, there is still a lack of theoretical research on the GSFE and work function of CoCrFeNi-based high-entropy alloys with different Mn. In general, the GSFE has been widely studied in characterization of the mechanical properties and brittle-to-ductile transition of alloys. The GSFE is considered to be a measure of energy penalty between two adjacent planes during shear deformation along a specific slip direction on a given slip plane. It represents the nature of the slip and involves stable and unstable stacking and twin fault energy. In particular, the intrinsic stacking fault energy (ISF) of the alloy can be calculated via transmission electron microscopy (TEM) [20,21] and X-ray powder diffraction (XRD) [22]. However, the unstable stacking fault energy (USF) cannot be measured by experiment, and the first principle calculation is an effective method by which to measure the USFE [23]. On the other hand, virtual crystal approximation (VCA) is considered a method by which to study the properties of solid solutions. The method uses “virtual” atoms inserted between atomic behaviors in the original compound to study crystals in primitive periodicity. This method makes the calculation simpler and the cost lower. Of course, previous work [24,25] has proved that VCA has good accuracy in some HEAs.

Therefore, based on the first-principle calculation, the theoretical models of Mn_x_CoCrFeNi HEAs are established via the VCA method in the present work. In order to study the effect of different Mn concentrations on slip and twin in Mn_x_CoCrFeNi HEAs, 13 close-packed (111) atomic layers are used to calculate GSFE, as well as USF, ISF, unstable twin-fault energy (UTF), and two-layers twinning stacking fault energy (TSF) parameters. More importantly, the elastic properties and electron density difference were systematically studied with the aim of further uncovering the nature of the strength and ductility of Mn_x_CoCrFeNi HEAs. Finally, the work functions of (111), (110), and (100) planes in Mn_x_CoCrFeNi HEAs are estimated, which reveals the analysis results in more details. Therefore, theoretical analysis was conducted on HEAs containing different proportions of Mn elements in this study. The influence of Mn element on the GSFE and other properties of CoCrFeNi-based HEAs can be obtained, which can provide useful guidance for the design of high-performance CoCrFeNi-based HEAs.

## 2. Methods and Details

### 2.1. First-Principles Calculations

All the first-principles calculations are performed using the Cambridge sequential total energy package (CASTEP) (MS 7.0) [26,27] based on density functional theory (DFT) [28,29]. The exchange correlation functional uses the Pardew–Burke–Ernzerhof (PBE) generalized gradient approximation (GGA) [30,31]. Clearly, the premise for simulation is geometry optimization (minimum energy of atoms at different volumes). Therefore, according to the termination of structural relaxation, the convergence parameters are selected. The convergence parameters were set as follows: total energy tolerance is 10^−5^ eV/atom; force tolerance is 0.03 eV/Å; maximum stress is 0.05 GPa; and maximum displacement is 0.001 Å. After the convergence test, the plane wave energy cutoff is 600 eV and the Monkhorst–Pack scheme [32,33] k-points set is 10 × 10 × 10 in the Brillouin zone. According to the selected setting, the error reaches less than 1% through the convergence of the total energy of the calculated model.

### 2.2. VCA Models

The construction of VCA unit cells is achieved by replacing real alloy atoms in the cell structure with “virtual” atoms, which are obtained from the weighted average technique of different alloy elements. The atomic percentages of each constituent atom within the cell of the Mn_x_CoCrFeNi high-entropy alloy are presented in Table 1.

Accurately determining the lattice constant of the unit cell is an essential prerequisite for predicting the properties of compounds in theoretical calculations. In this paper, the comparison between the optimized lattice constant and the lattice constant obtained from other calculation methods and experimental results is detailed in Table 2. In Table 2, SQS and CPA represent the estimation of lattice constants of high-entropy alloys using a special quasi random structure (SQS) and coherent-potential approximation (CPA), respectively. As shown in Table 2, the calculated lattice constant is consistent with other calculation methods and experimental results.

### 2.3. Stacking Fault Model and GSFE

**Figure 1:**
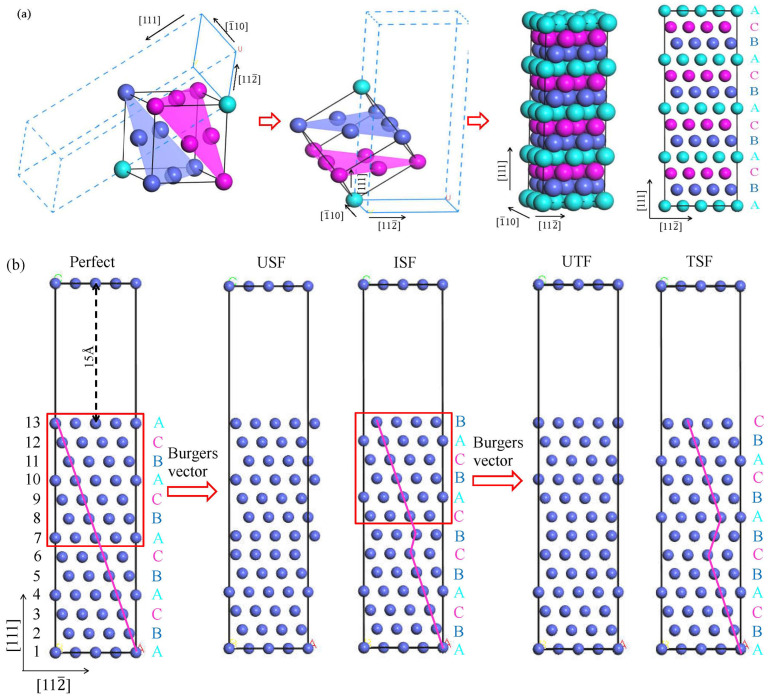
The computational cell used in the present work: (**a**) the atomic configuration of the FCC cell with stacking sequence A, B, and C; (**b**) the supercell consisting of 13 atomic layers of (111) plane.

The structure diagram of the FCC structural unit along [111], [$\bar{1}$10], and [11$\bar{2}$] directions is shown in Figure 1a. Based on the fact that dislocations in the FCC structure mainly occur on the (111) surface of dense packing, a supercell model with 13 closed-stacking (111) is constructed, in which the stacking sequence from bottom to top is “ABCABCABCABCA”. In Figure 1b, the GSFE value was obtained vis shear slip along [11$\bar{2}$] in the plane (111). In the first process, the upper 7–13 layers of atoms are sheared along the [11$\bar{2}$] direction. With the step size of 0.1b_p_, the unstable stacking fault (USF) is generated at the shear displacement of 0.0 to 0.5 b_p_, in which the Burgers vector $b_{p}=a_{0}/\sqrt{6}$. When the shear displacement increases to 1.0 b_p_, the structure becomes the ISF of ABCABCBCABCAB sequence. The second process is to move the upper 8–13 layers of atoms at the same distance along the [11$\bar{2}$] direction. When the shear displacement value increases to 1.0 b_p_, the structural sequence becomes ABCABCBABCABC, which is the TSF. The GSFE is calculated as follows [18]:

(1) $$\gamma_{GSFE}=\frac{1}{A}\left(E_{u}-E_{0}\right),$$

where *E_u_* is the total energy of the supercell after shear displacement, *E*_0_ is the total energy of the supercell without defect, and A is the area of the fracture plane. To avoid periodic atomic interactions, a 15 Å vacuum layer is added to the structure. After adequate convergence testing for the GSFE calculation, and considering the calculation efficiency, the plane wave energy cutoff and the k-points are set as 400 eV and 10 × 10 × 1, respectively.

### 2.4. Surface Energies

**Figure 2:**
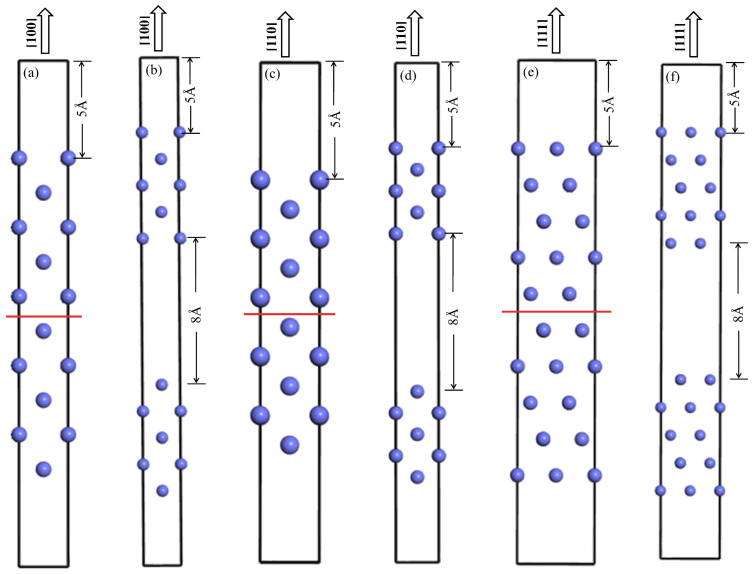
The perfect slab of (100), (110), and (111) planes containing 10 atomic layers with 5 Å vacuum layers in the bottom and top (**a**,**c**,**e**). Surface models of (100), (110), and (111) planes containing an 8 Å vacuum block in the center of the cell (**b**,**d**,**f**).

In order to better understand the surface properties of Mn_x_CoCrFeNi HEAs, the surface energies (γ_s_) of (100), (110), and (111) planes were calculated. Figure 2 illustrates the (100), (110), and (111) surface models of Mn_x_CoCrFeNi HEAs, which contain 10 atomic layers. A vacuum layer with a thickness of 5 Å is set on the bottom and top of the surface model to eliminate the influence of the interaction between periodic structures, as shown in Figure 2a,c,e. With the aim of avoiding the interaction between atoms in the slab, an 8 Å vacuum layer is set between the atomic layers of the slab model (Figure 2b,d,f). The surface energy can be calculated by the following formula [25,37]:

(2) $$\gamma_{s}=\frac{1}{2A}\left(E_{slab}-E_{Bulk}\right).$$

here, $E_{slab}$ is the total energy of the surface model. $E_{Bulk}$ is the total energy of the perfect supercell, and A is the surface area.

## 3. Results and Discussion

### 3.1. GSFE and Surface Energies

**Figure 3:**
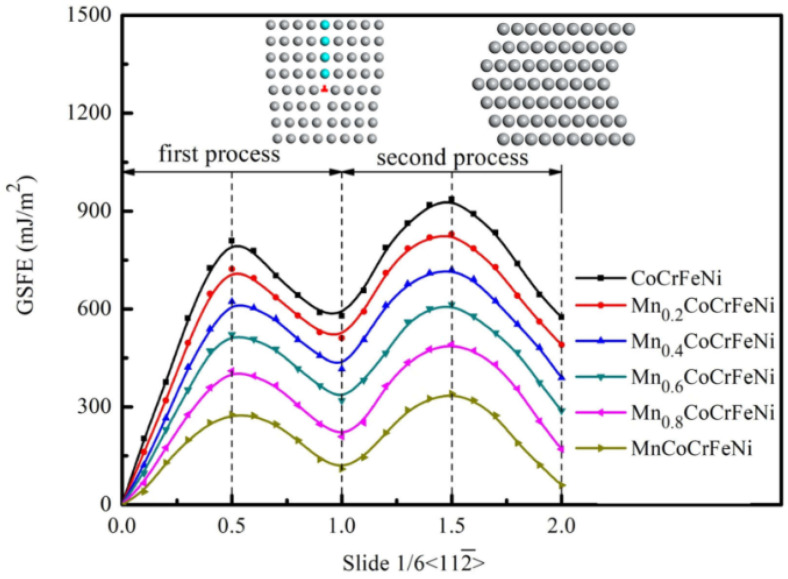
The calculated GSFE curves for Mn_x_CoCrFeNi HEAs.

Figure 3 and Table 1 exhibit the GSFE curves and related values of Mn_x_CoCrFeNi HEAs with different Mn concentrations, which were calculated based on first-principles. According to the Figure 3, with the increase in shear displacement along the direction of [11$\bar{2}$], the structure exhibits an energy barrier at the first highest energy point on the GSFE curve, which is called the unstable stacking fault energy (γ_usf_). This energy barrier is considered as the minimum energy or critical stress required for local dislocation nucleation. As the structure is further sheared, some dislocations begin to spread, and a stacking fault defect is created. The first minimum energy point on the GSFE curve is formed, and this energy is called intrinsic or stable stacking fault energy (γ_isf_). The second maximum energy point on the GSFE curve is called the unstable twin fault energy (γ_utf_). This energy is considered to be the minimum energy barrier to produce an extrinsic or twinning stacking fault. The second minimum energy point is defined as the extrinsic stacking fault energy or two-layer twinning stacking fault energy (2γ_tsf_). The GSFE calculation based on the first-principle separates the formation energy from any other related effects on total energy, so the calculated value may be more accurate than the experimental measurement value [18]. Van Swygenhoven et al. [38] found that only using SFE parameters as a criterion for judging the twinning and partial dislocation deformation mechanism of materials is not sufficient.

As can be seen from the Figure 3, with the increase in Mn concentration, the GSFE curves of Mn_x_CoCrFeNi HEAs gradually decreased. The USF energy decreases from 808.84 mJ/m^2^ to 276.17 mJ/m^2^ with increasing Mn content from 0.0 to 1.0. The results demonstrate that the alloy with higher Mn content easily forms dislocation due to its low nuclear dislocations resistance. The two-layer twinning SFE of HEAs was lower than that of the stable SFE. And the decrease degree of the two-layer twinning SFE was greater than that of stable SFE. When the Mn content was 0, the two-layer twinning SFE of HEAs (2γ_tsf_ = 574.71 mJ/m^2^) was lower than the stable SFE (γ_isf_ = 578.93 mJ/m^2^). When the Mn content was 1, the value of the two-layer twinning SFE is 60.36 mJ/m^2^, and the value of the stable SFE is 110.04 mJ/m^2^.

**Figure 4:**
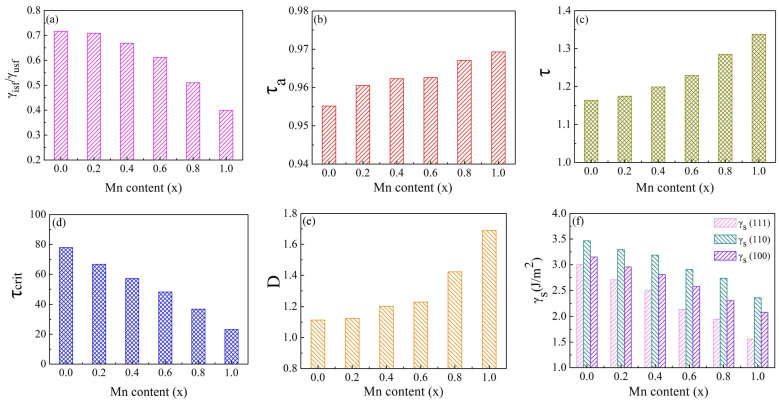
The calculated ratios of γ_isf_/γ_usf_ (**a**), twinnability for crack tip twinning τ_a_ (**b**), twinnability for grain boundary twinning τ (**c**), anticipated critical stress τ_crit_ (**d**), the Rice-criterion for ductility D (**e**), and the surface energies γ_s_ (**f**) for Mn_x_CoCrFeNi HEAs.

The ratio of ISF energy to USF energy (γ_isf_/γ_usf_) is often used to indicate the tendency of complete dislocation dissociation into partial dislocation. The lower the ratio, the greater the tendency of full dislocation dissociation. When the leading partial dislocations nucleate by overcoming the energy barrier γ_usf_, the tailing part needs to exceed this energy for nucleation. The critical stress for the nucleation of the trailing part is a function of (γ_usf_ − γ_isf_). The increased value of γ_usf_ − γ_isf_ is beneficial to the partial dislocation, and the stacking fault more likely to form. The difference between γ_usf_ and γ_isf_ in Mn_x_CoCrFeNi HEAs was large, implying that the deformation mechanism was only extended partial dislocation. Moreover, with the increase in Mn content, the difference value decreased gradually from the original 229.91 to 166.13 mJ/m^2^. The result displays that the tendency of stacking faults decreases with the increase in Mn content. As indicated in Figure 4a, the ratio of γ_isf_ and γ_usf_ in Mn_x_CoCrFeNi HEAs was larger, demonstrating that the energy barrier needed to form the trailing part was smaller, and it was difficult to produce stacking faults. In addition, Tadmor et al. [39] gave a standard by which the main deformation mechanism can be judged when mechanical twinning becomes an ideal crack tip. By this method, the tendency of partial dislocations to form total dislocations can be determined, resulting in dislocation-mediated slip or mechanical twins. Its expression is as follows.

(3) $$\delta_{usf}^{utf}=\gamma_{utf}-\gamma_{usf},$$

It is clear from Table 3 that the $\delta_{usf}^{utf}$ values of Mn_x_CoCrFeNi HEAs are positive, suggesting that the energy barrier for unstable twin formation (γ_utf_) is greater than the energy barrier for partial dislocation propagation (γ_usf_). It can be considered that the deformation mechanism of Mn_x_CoCrFeNi HEAs is not conducive to the transformation from dislocation to twins. This conclusion suggests that plastic deformation can be dominated by dislocation-mediated slip. However, with the increase in Mn concentration, its value decreases. It is hinted that mechanical twin can still be formed with the increase in Mn concentration. The existence of twin propagation starts from local dislocation or twin crystals, which is related to the γ_utf_/γ_usf_ ratio. If the ratio is low, twin deformation is more likely to occur. It is obvious in Table 1 that the ratio of γ_utf_/γ_usf_ has little correlation with the change in Mn concentration. For further study, Tadmor et al.’s [39] criterion was used to study the tendency of FCC metal forming mechanical twins. The formula is as follows:

(4) $$\tau_{a}=\left[1.136-0.151\frac{\gamma_{isf}}{\gamma_{usf}}\right]\sqrt{\frac{\gamma_{usf}}{\gamma_{utf}}},$$

Among them, 1.136 and 0.151 are the general coefficients of the FCC lattice. A higher $\tau_{a}$ value can achieve higher twinning tendency. According to Figure 4b, the $\tau_{a}$ values increase with the increase in the concentration of Mn in Mn_x_CoCrFeNi HEAs, but the change in $\tau_{a}$ was little. When the Mn concentration increases from 0.0 to 1.0, the $\tau_{a}$ value increases from 0.956 to 0.969, and the difference is only 0.013. It follows that the increase in Mn concentration can lead to twin formation, but the effect is not significant.

An alternative parameter τ, given by Asaro et al. [40], can reflect the competition between dislocation propagation and grain boundary source mechanical twins, and the expression is expressed as follows:

(5) $$\tau =\sqrt{\left(1+2\beta \right)\frac{\gamma_{usf}}{\gamma_{utf}}},$$

where $\beta =1-\gamma_{isf}/\gamma_{usf}$. When τ > 1, the twin phenomenon is considered to be the dominant mechanism, which is more conducive to complete dislocation. It can be observed from Figure 4c that all the τ values of HEAs are greater than 1, and the value gradually increases from 1.164 to 1.337 with the increase in Mn concentration. This shows that the increase in Mn concentration is more favorable for twin generation.

Kibey et al. [41] proposed a continuous, multi-scale method by which to predict twin stresses. The method uses the dislocation-based twin nucleation model to calculate the critical twin stress of GSFE in FCC metal, and the expression is as follows:

(6) $$\tau_{crit}=\frac{5}{18b_{twin}}\left(\gamma_{utf}+\frac{2\gamma_{tsf}+\gamma_{isf}}{2}\right)-\frac{2}{9b_{twin}}\left(\gamma_{usf}+\gamma_{isf}\right).$$

Twin phenomena in FCC structures are caused by shear along the direction of [11$\bar{2}$] on the (111) surface, so b_twin_ is defined as $a/\sqrt{6}$. According to Equation (6), $\tau_{crit}$ is determined by the four typical GSFE values (γ_isf_, γ_usf_, γ_utf_, and 2γ_tsf_), instead of only considering the correlation of γ_isf_. This method has been used to predict twin stresses of some FCC metals. When the $\tau_{crit}$ value decreases, the tendency to produce twins increases. Figure 4d demonstrates that the $\tau_{crit}$ value decreases gradually with the increase in Mn content, from 77.83 to 23.20. It is obvious that alloys with higher Mn content require less energy for critical twinning stress than alloys with lower Mn content, and twins are more likely to be produced.

The influence of different Mn contents on the ductility of Mn_x_CoCrFeNi HEAs can be analyzed using the Rice-criterion [42]. This analysis explains the competitive relationship between crack tip dislocation formation and crack cleavage. Its expression can be expressed as follows:

(7) $$D=0.3\frac{\gamma_{s}}{\gamma_{usf}},$$

where D is the ductility parameter and γ_s_ is the surface energy along the direction of [111]. When D > 1, the dissociation energy of the crack is greater than that of the dislocation nuclear energy. As a result, the alloy will exhibit ductile behavior. However, in the event of D < 0.3, the failure was caused by crack cleavage rather than dislocation slip. The calculated results listed in Figure 4e and Table 3 indicate that the D values of all alloys are greater than 1 and increase continuously with the increase in Mn concentration. The results show the Mn_x_CoCrFeNi HEAs’ ductility, and there was dislocation formation of the crack tip. Kivy M.B. et al. [17] studied the Rice-criterion ductilities for the alloy (D > 1), indicating that the display ductility of the alloy is consistent with the calculation results in this paper.

The calculated surface energies of Mn_x_CoCrFeNi HEAs (111), (110), and (100) surfaces are summarized in Figure 4f. It is obvious that in HEAs with different Mn concentrations, the close-packed (111) plane has the lowest surface energy. With the increase in Mn content, the surface energy of the three planes tends to decrease. When the x value changes from 0.1 to 1.0, in Mn_x_CoCrFeNi HEAs, the surface energy differences of (111), (110), and (100) is 1.447 J/m^2^, 1.102 J/m^2^, and 1.071 J/m^2^, respectively. Therefore, the surface energy of surface (111) is most susceptible to the increase in Mn concentration.

### 3.2. Elastic Properties

**Figure 5:**
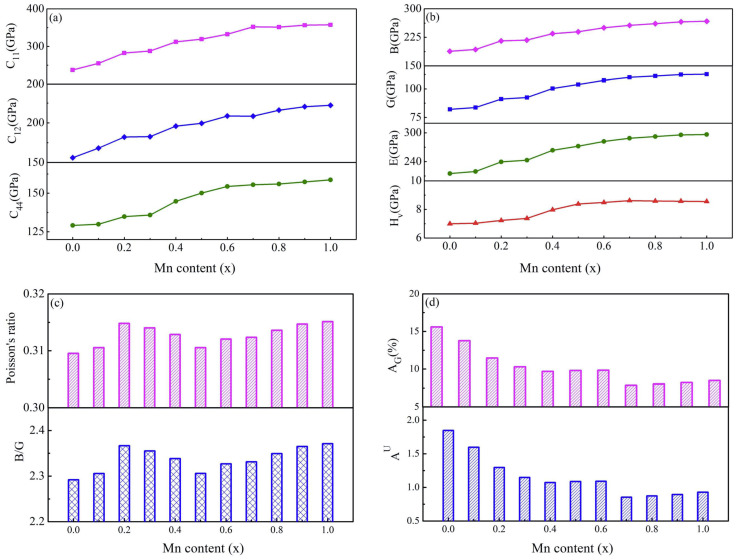
The effect of Mn concentration on Mn_x_CoCrFeNi HEAs elastic properties: (**a**) elastic constants; (**b**) bulk modulus, shear modulus, Young’s modulus, and Vickers hardness; (**c**) Poisson’s ratio and G/B; (**d**) anisotropy indexes A_G_ and A^U^.

With the goal of studying the elastic properties of Mn_x_CoCrFeNi HEAs, the elastic constants of the alloys were also calculated. Furthermore, the elastic modulus, Poisson’s ratio, and anisotropy of the alloys are analyzed. Mn_x_CoCrFeNi HEAs are a cubic crystal system, which has three independent elastic constants: C_11_, C_12_, and C_44_. The change in elasticity constant of Mn_x_CoCrFeNi HEAs with Mn concentration is expressed in Figure 5a. As displayed in the Figure 5a, the elastic constant of the alloy conforms to the mechanical criterion of the cubic crystal system [43]: C_11_ > 0; C_44_ > 0; C_11_−C_12_ > 0; C_11_ + 2C_12_ > 0. This is evidence that these alloys are mechanically stable. The three elastic constants gradually increase with the increase in Mn content. The change in C_11_ was more significant. As the value of x increases from 0 to 1, the value of C_11_ increases from 237.37 GPa to 357.48 GPa. Compared with C_11_, the variation trend of C_12_ and C_44_ is relatively gentle.

The bulk modulus (B), shear modulus (G), Young’s modulus (E), Vickers hardness (H_v_), and Poisson’s ratio (σ) of the alloy can be calculated according to the following formulae [44,45]:

(8) $$B_{H}=\frac{1}{2}\left(B_{V}+B_{R}\right),$$

(9) $$G_{H}=\frac{1}{2}\left(G_{V}+G_{R}\right),$$

(10) $$E=\frac{9B_{H}G_{H}}{\left(3B_{H}+G_{H}\right)},$$

(11) $$H_{v}=2{\left(G^{3}/B^{2}\right)}^{0.585}-3,$$

(12) $$\sigma =\frac{\left(3B_{H}-2G_{H}\right)}{\left[2\left(3B_{H}+G_{H}\right)\right]},$$

where subscripts V and R are the approximate values obtained by Voigt and Reuss, respectively. Subscript H represents the average values of these two approximations. The bulk modulus reflects the ability of the alloy to resist bulk deformation. Shear modulus can be used to measure the shear deformation resistance of the alloy. Young’s modulus can reflect the stiffness of the alloy. As can be seen from Figure 5b, with the increase in Mn concentration, the bulk modulus of HEAs increases gradually. The bulk modulus of the HEAs gradually increases from 188.23 GPa to 267.280 GPa. The change trend of Mn_x_CoCrFeNi HEAs’ shear modulus and Young’s modulus was consistent with the bulk modulus. When x is 0.7, the shear modulus and Young’s moduli of HEAs reach 109.951 GPa and 288.592 GPa, respectively. As the concentration of Mn continues to increase, the change trend tended to be gentle. The results demonstrate that along with the raise in Mn content, the volume deformation resistance, shear deformation resistance, and alloy stiffness of HEAs increases continuously. The Vickers hardness can be macroscopic reaction alloy hardness. It can be seen from Equation (11) that Vickers hardness is affected by bulk modulus and shear modulus. It can be observed from the Figure 5b that the Vickers hardness of the alloy increases with the increase in Mn concentration. When the value of x is 0.7, the Vickers hardness of the alloy achieves 8.615 GPa. After that, with the increase in Mn concentration, the Vickers hardness of the HEAs is almost unchanged. It is obvious that when Mn content is greater than 0.7, the Vickers hardness is no longer affected by Mn content.

The critical value of the Poisson’s ratio, which is 0.26, is often used to judge the ductility and brittleness of alloys. When the Poisson’s ratio is greater than 0.26, the compound is ductility. On the contrary, when the Poisson’s ratio is less than 0.26, the compound is brittle. It can be visualized in Figure 5c that the Poisson’s ratios of Mn_x_CoCrFeNi HEAs were all greater than 0.26, hinting that these alloys are ductile. B/G is another criterion for judging the brittleness and ductility of alloys. According to Pugh criterion [46], if the B/G value of an alloy is greater than 1.75, the material has ductility. In it is not, the material is brittle. It is presented in the Figure 5c that the values of B/G are all greater than 1.75, and their variation trend is similar to that of Poisson’s ratio. This is evidence that the alloys are ductile, which is consistent with the Poisson’s ratio analysis.

The anisotropy of crystal elasticity plays an important role in the study of macroscopic mechanical properties. Therefore, it is of great significance to study the influence of anisotropy of Mn_x_CoCrFeNi HEAs on the mechanical behavior of the alloy. The anisotropy index (A_B_ and A_G_) and the general anisotropy index (A^U^) can be calculated using the following formulae [47]:

(13) $$A_{B}=\frac{B_{V}-B_{R}}{B_{V}+B_{R}}\times 100\%,$$

(14) $$A_{G}=\frac{G_{V}-G_{R}}{G_{V}+G_{R}}\times 100\%,$$

(15) $$A^{U}=5\frac{G_{V}}{G_{R}}+\frac{B_{V}}{B_{R}}-6\geq 0.$$

When the values of A_B_, A_G_, and A^U^ are zero, this indicates that the crystal appears isotropic. On the contrary, when the anisotropy index is not equal to zero, the crystal shows anisotropy. The greater the deviation from 0, the greater the anisotropy. Since the crystal structure of Mn_x_CoCrFeNi HEAs had FCC cubic structure, the values of B_V_ and B_R_ were equal. The calculated bulk modulus anisotropy index is zero, implying that the bulk modulus of the HEAs is isotropic. The variation trend of A_G_ and A^U^ values with Mn content is revealed in Figure 5d. The trend of A_G_ and A^U^ is consistent. The A_G_ and A^U^ of the CoCrFeNi HEA are the largest, which are 15.589 and 1.847, respectively. With the increase in Mn content, their values decreased slightly. When the value of x reaches 0.7, the values of A_G_ and A^U^ decrease to the lowest values of 7.96 and 0.853. As the x value continues to increase, the values of A_G_ and A^U^ increase slightly, but the change is not significant.

**Figure 6:**
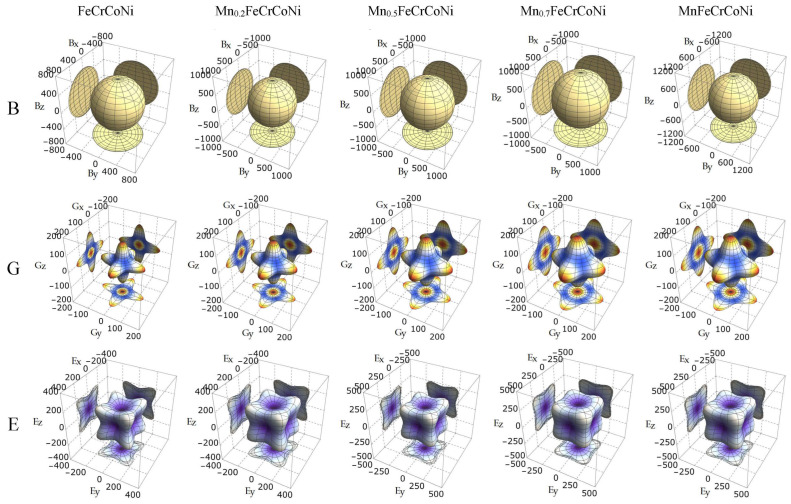
The anisotropic characteristics of the bulk modulus, shear modulus, and Young’s modulus of Mn_x_CoCrFeNi HEAs.

In order to more intuitively display the anisotropy of the alloy’s elastic modulus, the bulk modulus, shear modulus, and Young’s modulus of Mn_x_CoCrFeNi HEAs are drawn in different directions using the spherical coordinate method. The directional relation of each cubic crystal modulus can be determined via the following equations [48]:

(16) $$\frac{1}{B}=\left(S_{11}+2S_{12}\right)\left(l_{1}^{2}+l_{2}^{2}+l_{3}^{2}\right),$$

(17) $$\frac{1}{G}=S_{44}+4\left[(S_{11}-S_{12})-\frac{S_{44}}{2}\right]\left(l_{1}^{2}l_{2}^{2}+l_{2}^{2}l_{3}^{2}+l_{1}^{2}l_{3}^{2}\right),$$

(18) $$\frac{1}{E}=S_{11}-2\left(S_{11}-S_{12}-\frac{S_{44}}{2}\right)\left(l_{1}^{2}l_{2}^{2}+l_{2}^{2}l_{3}^{2}+l_{1}^{2}l_{3}^{2}\right).$$

where S_ij_ denotes the elastic compliance constants; l_1_, l_2_, and l_3_ denote the directional cosines; and $l_{1}=\sin\theta \cos\phi ;\;l_{2}=\sin\theta \sin\phi ;\;l_{3}=\cos\theta$. When the 3D diagram is spherical, this means that the alloy is isotropic. Conversely, when the 3D diagram deviates from the sphere, the alloy is anisotropic. The greater the deviation, the greater the anisotropy. As illustrated in Figure 6, the anisotropic 3D diagrams of the bulk modulus, shear modulus and Young’s modulus of CoCrFeNi, Mn_0.2_CoCrFeNi, Mn_0.5_CoCrFeNi, Mn_0.7_CoCrFeNi, and MnCoCrFeNi are drawn, respectively. In Figure 6, the 3D figure of the bulk modulus is single in color and spherical, demonstrating that the bulk moduli of these HEAs are isotropic. The shear modulus and Young’s modulus are all deviated from the sphere, meaning that the shear modulus and Young’s modulus are anisotropic. The 3D diagram of the shear modulus of CoCrFeNi deviates most from the sphere, which clearly indicates that the shear modulus anisotropy of CoCrFeNi is the most severe. On the contrary, the deviation of the 3D diagram of the shear modulus of Mn_0.7_CoCrFeNi from the sphere is relatively small, so the shear modulus anisotropy of Mn_0.7_CoCrFeNi is small. The 3D diagram of the Young’s modulus indicates a similar trend to that of the shear modulus. These results are consistent with the previous analysis results of A_B_ and A_G_.

### 3.3. Electronic Properties

**Figure 7:**
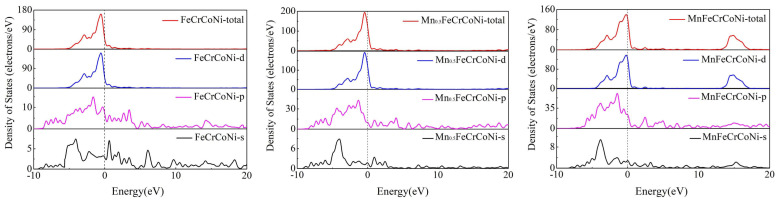
Total and partial density of Mn_x_CoCrFeNi HEAs (x = 0, 0.5, and 1).

In order to study the electronic properties of Mn_x_CoCrFeNi high-entropy alloys with different Mn concentrations, the density of states and charge density difference of CoCrFeNi, Mn_0.5_CoCrFeNi, and MnCoCrFeNi are also calculated. The total and partial density of states is plotted from −15 to 40 eV. The Fermi energy level (E_f_) at 0 eV is marked with a dashed line. As can be seen from Figure 7, the d-orbital electrons have a strong value compared with the s-orbital and p-orbital electrons in the whole range. The TDOS is mainly contributed by the d-orbital, and the peak of the TDOS is mainly distributed in the range of −5–2 eV near the Fermi level. In the case of x = 0, 0.5, and 1.0, the total densities of states at Fermi level are 45.05, 120.44, and 124.69, respectively. Obviously, the total density of states increases as the value of x increases. It can be inferred that the stability of the HEAs decrease with the increase in Mn concentration.

**Figure 8:**
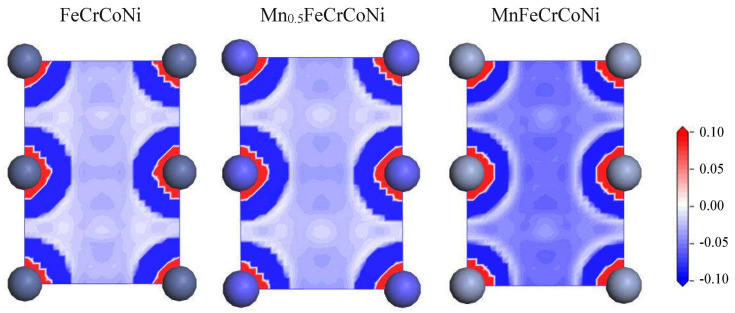
The electron density difference of the (110) plane in Mn_x_CoCrFeNi HEAs (x = 0, 0.5, and 1).

In the VCA model, all atoms in a cell are the same, so the calculation of charge density difference is related to the atomic density difference in FCC cell and the VCA model. In the end, the differences in electron density of Mn_x_CoCrFeNi HEAs are simply compared. Figure 8 shows a two-dimensional slice of the charge density difference of (110) plane in Mn_x_CoCrFeNi (x = 0, 0.5, and 1) HEAs. The plotting range of charge density difference is from −0.1 to 0.1 e/Å^3^. Electron loss is represented in blue and electron accumulation in red. In Figure 8, is evident that with the increase in Mn content, the degree of charge transfer gradually decreases. The weakening of atomic bonding leads to the instability of Mn_x_CoCrFeNi, which can increase the possibility of dislocation.

### 3.4. Work Function

**Figure 9:**
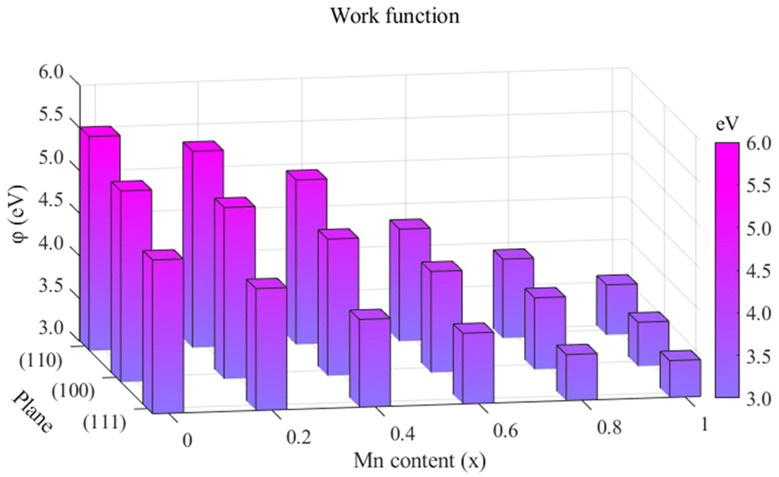
The effect of Mn concentration on work functions of Mn_x_CoCrFeNi HEAs (100), (110), and (111) planes.

Generally, the electron work function (EWF) is the minimum amount of energy required for an electron in a metal to move to its surface at the Fermi level. It is one of the basic electronic properties of metals, depending on their composition and surface conditions. It fundamentally reflects the interaction between atoms and is directly related to the physical properties. In order to further understand the properties of Mn_x_CoCrFeNi HEAs, the degrees of EWF of low exponent surfaces (100), (110), and (111) were calculated. The calculated trend of the EWF of Mn_x_CoCrFeNi HEAs with a change in Mn concentration is drawn in Figure 9. It can be seen that the EWF of surface (110) is the largest, followed by surface (100) and surface (111). With the increase in Mn concentration, the EWF of (111), (110), and (100) decreases continuously. As the EWF decreases, the atomic bonds weaken. Therefore, with the raise of Mn concentration, dislocations are more likely to occur, which is in accordance with the results of previous GSFE analysis. The research results of Shi Y.Z. et al. [15] proved that with the decrease in the work function, the alloy system containing high passivation elements (Cr, Ni, Mo, Ti, etc.) has higher corrosion resistance. There were passivation elements Cr and Ni in Mn_x_CoCrFeNi HEAs, and the EWF gradually decreased. It can be inferred that the corrosion resistance of Mn_x_CoCrFeNi HEAs heightens when accompanied by Mn concentration. The study of Lu H. et al. [49] demonstrated that the Young’s modulus and hardness would increase with the increase in EWF and the strengthening of atomic bonds. It is worth noting that the work functions of Mn_x_CoCrFeNi HEAs have different trends with respect to the Young’s modulus and hardness.

## 4. Conclusions

In this paper, the stacking fault energy, elastic properties, electronic properties, and work function of the Mn_x_CoCrFeNi high-entropy alloy are analyzed using density functional theory. The calculated results can be summarized as follows.

The results of the GSFE curves demonstrate that the alloy with higher Mn content has lower anti-dislocation nucleation ability and easily forms dislocation. The relative barrier heights ($\delta_{usf}^{utf}$) of the HEAs are all positive, which indicates that the deformation mechanism of the HEAs is not conducive to the transformation from dislocation to twin. The results of the GSFE and surface energies suggest that plastic deformation is controlled by dislocation-mediated slip. However, with the increase in Mn concentration, the value of $\delta_{usf}^{utf}$ decreases, which suggests that mechanical twinning can still be formed in the alloy. Based on the Rice-criterion, it is inferred that the Mn_x_CoCrFeNi HEAs are ductile and have the formation of crack tip dislocation.

With the increase in Mn concentration, the bulk modulus, shear modulus, and Young’s modulus of Mn_x_CoCrFeNi HEAs tend to increase. It can be concluded that with the enhancement of Mn concentration, the deformation resistance, shear resistance, and stiffness of the HEAs are improved. The density of states of Mn_x_CoCrFeNi (x = 0, 0.5, and 1) HEAs at the Fermi level decreases with the increase in x, which indicates that the stability of the alloy decreases. The calculation of charge density difference demonstrates that the degree of charge transfer decreases gradually. This phenomenon will weaken the atomic bond and lead to an increase in the instability of the alloy, thus increasing the possibility of dislocation. However, the work function variation trend of Mn_x_CoCrFeNi HEAs with different Mn concentrations is different from the Young’s modulus.

## Figures and Tables

**Table 1:** Chemical compositions (in at.%) of Mn_x_CoCrFeNi HEAs.

HEAs	Mn (at.%)	Co (at.%)	Cr (at.%)	Fe (at.%)	Ni (at.%)
CoCrFeNi	0	25.0	25.0	25.0	25.0
Mn_0.2_CoCrFeNi	2.4	24.4	24.4	24.4	24.4
Mn_0.4_CoCrFeNi	9.2	22.7	22.7	22.7	22.7
Mn_0.6_CoCrFeNi	13.2	21.7	21.7	21.7	21.7
Mn_0.8_CoCrFeNi	16.8	20.8	20.8	20.8	20.8
MnCoCrFeNi	20.0	20.0	20.0	20.0	20.0

**Table 2:** Calculated lattice parameter (in Å) of HEAs in the VCA model compared with previous experiment (Expt.).

HEAs	a	$a_{SQS}$	$a_{CPA}$	$a_{Expt.}$
CoCrFeNi	3.415	3.540 [34]	3.529 [35]	3.575 [34]
MnCoCrFeNi	3.517	3.540 [34]	3.529 [35]	3.597 [34]
				3.59 [36]

**Table 3:** The calculated unstable γ_usf_ (mJ/m^2^), stable γ_isf_ (mJ/m^2^), unstable twinning γ_utf_ (mJ/m^2^), twinning 2γ_tsf_ (mJ/m^2^), energy barrier height $\delta_{usf}^{utf}$, γ_isf_/γ_usf_, γ_utf_/γ_usf_ parameter, Rice-criterion D and surface energies of (100), (110) and (111) planes (J/m^2^) in Mn_x_CoCrFeNi HEAs.

HEAs(x)	γ_usf_	γ_isf_	γ_utf_	2γ_tsf_	$\delta_{usf}^{utf}$	γ_isf_/γ_usf_	γ_utf_/γ_usf_	D	γ_s_(111)	γ_s_(110)	γ_s_(100)
0.0	808.84	578.93	935.74	574.71	126.90	0.716	1.157	1.113	3.002	3.465	3.150
0.2	722.68	511.42	829.74	490.12	107.06	0.708	1.148	1.123	2.706	3.292	2.955
0.4	622.57	416.32	720.54	389.04	97.96	0.669	1.157	1.202	2.495	3.187	2.807
0.6	521.78	319.25	613.32	288.60	91.55	0.612	1.175	1.229	2.137	2.909	2.576
0.8	410.07	209.17	491.73	169.97	81.66	0.510	1.199	1.423	1.945	2.737	2.307
1.0	276.17	110.04	340.21	60.36	64.05	0.398	1.232	1.689	1.555	2.363	2.080

## Data Availability

The data that support the findings of this study are available from the corresponding author upon reasonable request.

## References

[1] Yeh J.W., Chen S.K., Lin S.J., Gan J.Y., Chin T.S., Shun T.T., Tsau C.H., Chan S.Y. (2004). Nanostructured high-entropy alloys with multiple principal elements: Novel alloy design concepts and outcomes. Adv. Eng. Mater..

[2] Liu W.H., Lu Z.P., He J.Y., Luan J.H., Wang Z.J., Liu B., Liu Y., Chen M.W., Liu C.T. (2016). Ductile CoCrFeNiMo_x_ high entropy alloys strengthened by hard intermetallic phases. Acta Mater..

[3] Tao L., Sun M., Zhou Y., Luo M., Lv F., Li M., Zhang Q., Gu L., Huang B., Guo S. (2022). A general synthetic method for high-entropy alloy subnanometer ribbons. J. Am. Chem. Soc..

[4] Tsai K.Y., Tsai M.H., Yeh J.W. (2013). Sluggish diffusion in Co-Cr-Fe-Mn-Ni high-entropy alloys. Acta Mater..

[5] Cao B., Wang C., Yang T., Liu C. (2020). Cocktail effects in understanding the stability and properties of face-centered-cubic high-entropy alloys at ambient and cryogenic temperatures. Scr. Mater..

[6] Li J., Chen H., Fang Q., Jiang C., Liu Y., Liaw P.K. (2020). Unraveling the dislocation–precipitate interactions in high-entropy alloys. Int. J. Plast..

[7] Ding Q., Zhang Y., Chen X., Fu X., Chen D., Chen S., Gu L., Wei F., Bei H., Gao Y. (2019). Tuning element distribution, structure and properties by composition in high-entropy alloys. Nature.

[8] Ma E. (2020). Unusual dislocation behavior in high-entropy alloys. Scr. Mater..

[9] Qin G., Chen R., Liaw P.K., Gao Y., Li X., Zheng H., Wang L., Su Y., Guo J., Fu H. (2019). A novel face-centered-cubic high-entropy alloy strengthened by nanoscale precipitates. Scr. Mater..

[10] Yan X.H., Zhang Y. (2020). Functional properties and promising applications of high entropy alloys. Scr. Mater..

[11] Cantor B., Chang I.T.H., Knight P., Vincent A.J.B. (2004). Microstructural development in equiatomic multicomponent alloys. Mater. Sci. Eng. A.

[12] Li Z., Pradeep K.G., Deng Y., Raabe D., Tasan C.C. (2016). Metastable high-entropy dual-phase alloys overcome the strength-ductility trade-off. Nature.

[13] Sun X., Zhang H., Lu S., Ding X.D., Wang Y.Z., Vitos L. (2017). Phase selection rule for Al-doped CrMnFeCoNi high-entropy alloys from first-principles. Acta Mater..

[14] Zhang H., Sun X., Lu S., Dong Z., Ding X., Wang Y., Vitos L. (2018). Elastic properties of Al_x_CrMnFeCoNi (0 ≤ x ≤ 5) high-entropy alloys from ab initio theory. Acta Mater..

[15] Shi Y.Z., Collins L., Feng R., Zhang C., Balke N., Liaw P.K., Yang B. (2018). Homogenization of Al_x_CoCrFeNi high-entropy alloys with improved corrosion resistance. Corros. Sci..

[16] Zhang J., Xiong K., Huang L., Xie B., Ren D., Tang C., Feng W. (2023). Effect of Doping with Different Nb Contents on the Properties of CoCrFeNi High-Entropy Alloys. Materials.

[17] Kivy M.B., Zaeem M.A. (2017). Generalized stacking fault energies, ductilities, and twinnabilities of CoCrFeNi-based face-centered cubic high entropy alloys. Scr. Mater..

[18] Achmad T.L., Fu W., Chen H., Zhang C., Yang Z.G. (2016). First-principles calculations of generalized-stacking-fault-energy of Co-based alloys. Comput. Mater. Sci..

[19] Liu S.F., Wu Y., Wang H.T., He J.Y., Liu J.B., Chen C.X., Liu X.J., Wang H., Lu Z.P. (2018). Stacking fault energy of face-centered-cubic high entropy alloys. Intermetallics.

[20] Lu J., Hultman L., Holmström E., Antonsson K.H., Grehk M., Li W., Vitos L., Golpayegani A. (2016). Stacking fault energies in austenitic stainless steels. Acta Mater..

[21] Xu X., Liu P., Tang Z., Hirata A., Song S., Nieh T., Liaw P., Liu C., Chen M. (2018). Transmission electron microscopy characterization of dislocation structure in a face-centered cubic high-entropy alloy Al_0.1_CoCrFeNi. Acta Mater..

[22] Ullrich C., Eckner R., Krüger L., Martin S., Klemm V., Rafaja D. (2016). Interplay of microstructure defects in austenitic steel with medium stacking fault energy. Mater. Sci. Eng. A.

[23] Zhang Y., Guo J., Chen J., Wu C., Kormout K.S., Ghosh P., Zhang Z. (2019). On the stacking fault energy related deformation mechanism of nanocrystalline Cu and Cu alloys: A first-principles and TEM study. J. Alloys Compd..

[24] Mayahi R. (2020). An investigation concerning generalized stacking fault behavior of AlCo_x_CrFeNi (0.25 ≤ x ≤ 2) high entropy alloys: Insights from first-principles study. J. Alloys Compd..

[25] Zhao Q., Li J., Fang Q., Feng H. (2019). Effect of Al solute concentration on mechanical properties of Al_x_FeCuCrNi high entropy alloys: A first-principles study. Phys. B.

[26] Nong Z.S., Lei Y.N., Zhu J.C. (2018). Wear and oxidation resistances of AlCrFeNiTi-based high entropy alloys. Intermetallics.

[27] Pan Y., Pu D. (2020). First-principles investigation of oxidation behavior of Mo_5_SiB_2_. Ceram. Int..

[28] Mitro S.K., Hossain K.M., Majumder R., Hasan M.Z. (2021). Effect of the negative chemical pressure on physical properties of doped perovskite molybdates in the framework of DFT method. J. Alloys Compd..

[29] Vu V.T., Lavrentyev A.A., Gabrelian B.V., Parasyuk O.V., Ocheretova V.A., Khyzhun O.Y. (2018). Electronic structure and optical properties of Ag_2_HgSnSe_4_: First-principles DFT calculations and X-ray spectroscopy studies. J. Alloys Compd..

[30] Peivaste I., Alahyarizadeh G., Minuchehr A., Aghaie M. (2018). Comparative study on mechanical properties of three different SiCpolytypes (3C, 4H and 6H) under high pressure: First-principle calculations. Vacuum.

[31] Chen J.Y., Zhang X.D., Ying C.H., Ma H., Li J., Wang F., Guo H. (2020). The influence of vacancy defects on elastic and electronic properties of TaSi (5/3) desilicides from a first-principles calculations. Ceram. Int..

[32] Pang X.Z., Yang W.C., Yang J.B., Pang M.J., Zhan Y.Z. (2018). Atomic structure, stability and electronic properties of S(Al_2_CuMg)/Al interface: A first-principles study. Intermetallics.

[33] Chen S., Pan Y., Wang D., Deng H. (2020). Structural stability and electronic and optical properties of bulk WS_2_ from first-principles investigations. Mat. Sci. Eng. B-Solid..

[34] Zaddach A.J., Niu C., Koch C.C., Irving D.L. (2013). Mechanical properties and stacking fault energies of NiFeCrCoMn high-entropy alloy. Jom.

[35] Li X., Irving D.L., Vitos L. (2018). First-principles investigation of the micromechanical properties of fcc-hcp polymorphic high-entropy alloys. Sci. Rep..

[36] Cantor B. (2014). Multicomponent and high entropy alloys. Entropy.

[37] Udagawa Y., Yamaguchi M., Abe H., Sekimura N., Fuketa T. (2010). Ab initio study on plane defects in zirconium-hydrogen solid solution and zirconium hydride. Acta Mater..

[38] Swygenhoven H.V., Derlet P.M., Frøseth A.G. (2004). Stacking fault energies and slip in nanocrystalline metals. Nat. Mater..

[39] Tadmor E.B., Hai S. (2003). A Peierls criterion for the onset of deformation twinning at a crack tip. J. Mech. Phys. Solids.

[40] Asaro R.J., Suresh S. (2005). Mechanistic models for the activation volume and rate sensitivity in metals with nanocrystalline grains and nano-scale twins. Acta Mater..

[41] Kibey S., Liu J.B., Johnson D.D., Sehitoglu H. (2007). Predicting twinning stress in fcc metals: Linking twin-energy pathways to twin nucleation. Acta Mater..

[42] Rice J.R. (1992). Dislocation nucleation from a crack tip an analysis based on the Peierls concept. J. Mech. Phys. Solids.

[43] Wen Z.Q., Zhao Y.H., Hou H., Wang B., Han P.D. (2017). The mechanical and thermodynamic properties of Heusler compounds Ni_2_XAl (X = Sc, Ti, V) under pressure and temperature: A first-principles study. Mater. Des..

[44] Sun F., Zhang G., Liu H., Xu H., Fu Y., Li D. (2021). Effect of transition-elements substitution on mechanical properties and electronic structures of B2-AlCu compounds. Results Phys..

[45] Chen X.Q., Niu H., Li D., Li Y. (2011). Modeling hardness of polycrystalline materials and bulk metallic glasses. Intermetallics.

[46] Pugh S.F. (1954). XCII. Relations between the elastic moduli and the plastic properties of polycrystalline pure metals. Philos. Mag..

[47] Sun F., Zhang G., Ren X., Wang M., Xu H., Fu Y., Tang Y., Li D. (2020). First-principles studies on phase stability, anisotropic elastic and electronic properties of Al-La binary system intermetallic compounds. Mater. Today Commun..

[48] Nye J.F. (1985). Physical Properties of Crystals: Their Representation by Tensors and Matrices.

[49] Lu H., Liu Z., Yan X., Li D., Parent L., Tian H. (2016). Electron work function-a promising guiding parameter for material design. Sci. Rep..

